# Transcriptome signature of miRNA-26b KO mouse model suggests novel targets

**DOI:** 10.1186/s12863-021-00976-1

**Published:** 2021-06-30

**Authors:** Emiel P. C. van der Vorst, Mario A. A. Pepe, Linsey J. F. Peters, Markus Haberbosch, Yvonne Jansen, Ronald Nauman, Georgios T. Stathopoulos, Christian Weber, Kiril Bidzhekov

**Affiliations:** 1 Institute for Cardiovascular Prevention (IPEK), Ludwig-Maximilians-Universität (LMU) München, Munich, Germany; 2 DZHK (German Centre for Cardiovascular Research), partner site Munich Heart Alliance, Munich, Germany; 3 Interdisciplinary Center for Clinical Research (IZKF), Institute for Molecular Cardiovascular Research (IMCAR), RWTH Aachen University, Aachen, Germany; 4 Department of Pathology, Cardiovascular Research Institute Maastricht (CARIM), Maastricht University, Maastricht, the Netherlands; 5 Helmholtz Center Munich, Munich, Germany; 6 MPI of Molecular Cell Biology and Genetics, Dresden, Germany; 7 Department of Biochemistry, Cardiovascular Research Institute Maastricht (CARIM), Maastricht University, Maastricht, the Netherlands; 8 Munich Cluster for Systems Neurology (SyNergy), Munich, Germany

**Keywords:** MicroRNA-26b, Knock-out mouse model, Next generation sequencing, Cancer, Neurological disorders, Cardiovascular disease, Thermogenesis, Allergic reactions

## Abstract

**Background:**

MicroRNAs (miRNAs) are short (20–24 nt) non-coding RNAs that are involved in post-transcriptional regulation of gene expression in multicellular organisms by affecting both the stability and translation of mRNAs. One of the miRNAs that has been shown to play a role in various pathologies like cancer, neurological disorders and cardiovascular diseases is miRNA-26b. However, these studies only demonstrated rather ambiguous associations without revealing a causal relationship. Therefore, the aim of this study is to establish and validate a mouse model which enables the elucidation of the exact role of miRNA-26b in various pathologies.

**Results:**

A miRNA-26b-deficient mouse model was established using homologous recombination and validated using PCR. miRNA-26b-deficient mice did not show any physiological abnormalities and no effects on systemic lipid levels, blood parameters or tissue leukocytes. Using next generation sequencing, the gene expression patterns in miRNA-26b-deficient mice were analyzed and compared to wild type controls. This supported the already suggested role of miRNA-26b in cancer and neurological processes, but also revealed novel associations of miRNA-26b with thermogenesis and allergic reactions. In addition, detailed analysis identified several genes that seem to be highly regulated by miRNA-26b, which are linked to the same pathological conditions, further confirming the role of miRNA-26b in these pathologies and providing a strong validation of our mouse model.

**Conclusions:**

miRNA-26b plays an important role in various pathologies, although causal relationships still have to be established. The described mouse model of miRNA-26b deficiency is a crucial first step towards the identification of the exact role of miRNA-26b in various diseases that could identify miRNA-26b as a promising novel diagnostic or even therapeutic target in a broad range of pathologies.

**Supplementary Information:**

The online version contains supplementary material available at 10.1186/s12863-021-00976-1.

## Background

About 25 years after the discovery of the first microRNA (miRNA) [1], the field of miRNA research is flourishing as many thousands of miRNAs have already been discovered in more than 140 species, including more than 1900 in humans [2]. Although, the exact function of several miRNA remains unknown, many have already been implicated in a variety of physiological and pathological processes [3].

MiRNAs are single-stranded, small (20 to 24 nt), non-coding RNAs that target mRNAs with complementary sequences, thereby inhibiting the expression of their target genes at the post-transcriptional level or causing cleavage of the target mRNA [4]. Recognition of their mRNAs targets occurs by binding to the 3′ untranslated regions (UTR), where multiple miRNA molecules can simultaneously bind to the same mRNA and additionally one miRNA can also exert its effects on multiple target mRNAs. This results in a complex post-transcriptional regulatory network which plays a role in various important cellular and biological processes [5].

One of the miRNAs that has been demonstrated over the last years to play a crucial role in various pathologies is miRNA-26b. Most studies focused on the role of miRNA-26b-5p in carcinogenesis, showing that this miRNA is a critical regulator that acts either as an oncogene or tumor suppressor gene. For example, miRNA-26b-5p is down-regulated in a variety of malignant tumors, like hepatocellular carcinoma [6], nasopharyngeal carcinoma [7] and breast cancer [8]. However, elevated expression of miRNA-26b-5p has been demonstrated in pituitary tumors [9] and bladder cancer [10]. Besides an ambiguous role in carcinogenesis, recently it has also been shown that miRNA-26b-5p is linked to cognitive diseases. miRNA-26b-5p expression attenuated microglial activation, neurotoxicity and thereby vascular cognitive impairment [11]. However, in a mouse model for Alzheimer’s disease, miRNA-26b-5p expression levels were upregulated upon disease progression [12], again demonstrating a complex and ambiguous role for this miRNA. Furthermore, miRNA-26b-5p has been demonstrated to play a crucial role in hepatitis and liver fibrogenesis [13, 14]. Finally, in a previous study we could demonstrate that miRNA-26b-5p is also associated with atherosclerosis, the main cause of cardiovascular diseases (CVDs), as the miRNA-26b-5p expression was substantially upregulated in human plaque tissue compared to tissue from healthy *A. mammaria interna* [15]. All of these studies clearly demonstrate a strong association of miRNA-26b-5p expression with various pathologies, although these interactions seem to be very ambiguous and clear causal relationships have not yet been established.

Based on the important role that miRNA-26b seems to play in a wide variety of pathologies and the lack of causality in previous studies, the aim of the current study is to generate and phenotype a miRNA-26b knock-out mouse model that lacks both the miRNA-26b-3p as well as the miRNA26-5p (from now on called “miRNA-26b”). This model could be widely applicable to clarify the exact role of miRNA-26b in various pathologies. Furthermore, next generation sequencing was employed to elucidate the gene expression patterns in the aortic vessel to elucidate novel or validate proposed targets of miRNA-26b.

## Results

### Generation of miR-26b deficient mice

The coding region of the mouse *miRNA-26b* gene is situated in intron 4 of the *Ctdsp1* gene on chromosome 1 (Chr 1: 74,391,509-74,397,285 fw). The full sequence of this region has already been described (NCBI, NR_029743.1). Genomic clones containing the *miRNA-26b* gene and the genomic region in 5′ and 3′ direction were isolated and partially sequenced.

**Fig. 1:**
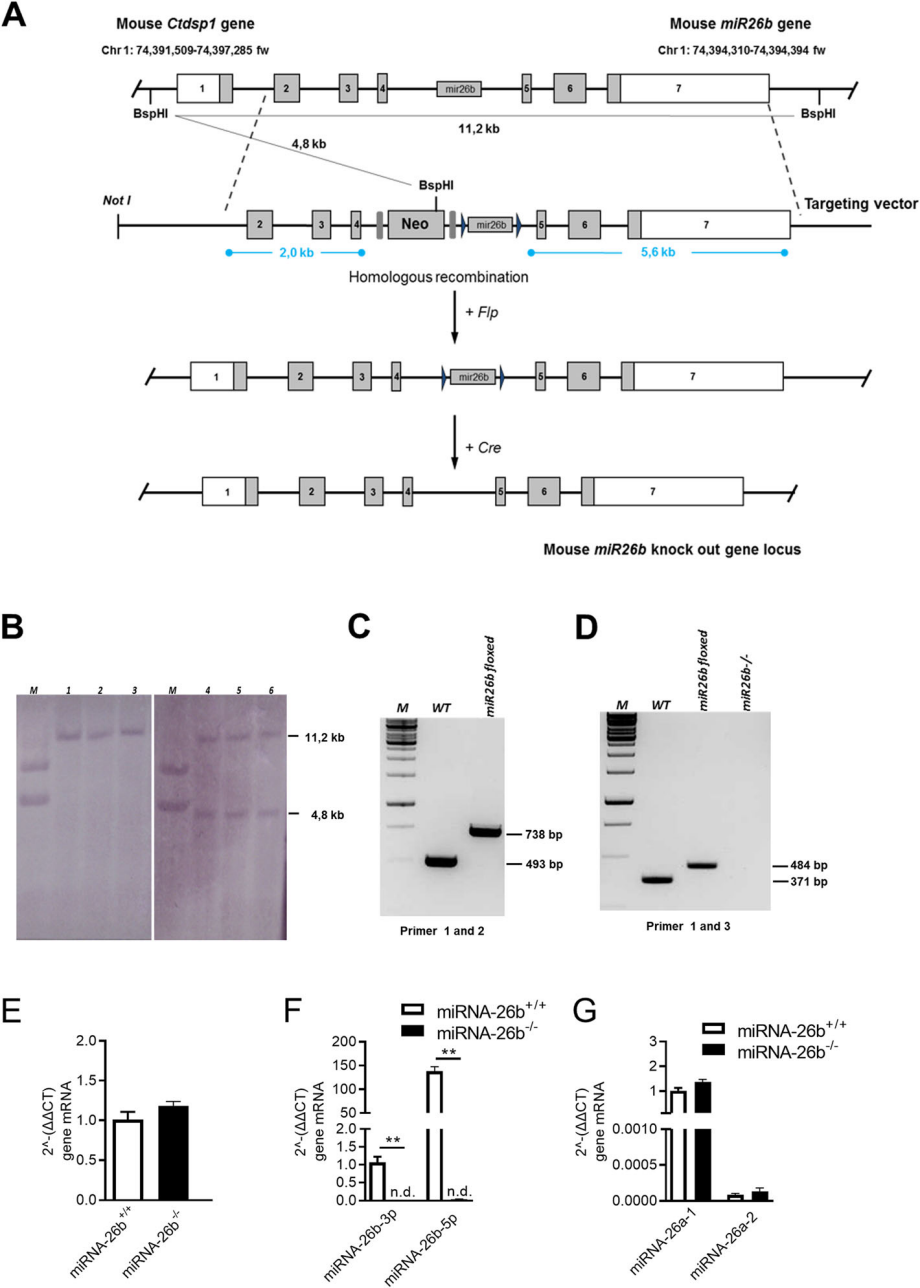
Generation and validation of miRNA-26b^−/−^ mice. **a** Schematic diagram of the generation of miR26b knockout mice. The exon-intron structure of the mouse host gene (*Ctdsp1*) locus is shown at the top. The targeting vector has a 2.0 kb 5′ arm including exon 2,3,4 and intron 4–5, a Neo selection cassette is flanked by FRT sites (gray bars). The loxP sites (black triangles) flanking *miR26b*. The 3′ recombination arm spanned 5.6 kb from the gDNA. **b** Southern blots show control (wild-type (1, 2, 3)) and correctly targeted heterozygote embryonic stem cell clones (4, 5,6) that gave expected hybridization patterns. **c** Homologous recombination was proved by primers spanning the miR26b floxed region. **d** Genotyping strategy based on primers identifying the wild-type, miR-26b floxed and miR-26b^−/−^ condition. **e-g** Relative mRNA expression measured by real time PCR of *Ctdps1* (**e**), *miRNA-26b-3p* and *miRNA-26b-5p* (**f**), *miRNA-26a-1* and *miRNA-26a-2* (**g**). Data represent mean ± SEM, as analyzed by Mann-Whitney test

In order to generate miRNA-26b deficient mice, homologous recombination in embryonic stem (ES) cells was used to disrupt the *miRNA-26b* encoding part consisting of 160 bp, while concomitantly preserving the splicing sites of the host *Ctdsp1* gene. The targeting vector was designed to conditionally replace the aimed region with the neomycin resistance gene (Fig. 1a). Homologous recombination in independent ES cell clones (R1/E(129/Sv) was confirmed by Southern blotting (Fig. 1b) and subsequently injected into blastocysts to generate chimeras. Male chimeras from two different ES cell clones were crossed with C57BL/6 females to establish strains with a mixed genetic background (129/Sv-C57BL/6) and mice that are heterozygous for the mutated allele. Homologous recombination in the offspring was confirmed by PCR (Fig. 1c).

The absence of miRNA-26b in the knock-out mice was confirmed after tamoxifen injection by conventional PCR with genomic DNA originating from P7 animals and primers binding into the targeted region. No *miRNA-26b* product was amplified from knock-out tissue, while a band of the expected size was amplified from wild-type and floxed tissue (Fig. 1d). Real time primers specific for *Ctdsp1* gene were used to ensure that expression of the host gene was not deregulated/interrupted (Fig. 1e). Furthermore, real time PCR confirmed the complete lack of both the miRNA-26b-3p as well as the miRNA-26b-5p product (Fig. 1f), while no compensatory effects by the closely related miRNA-26a-1 and miRNA-26a-2 could be observed (Fig. 1g).

### MiRNA-26b^−/−^ does not show any physiological phenotype

**Fig. 2:**
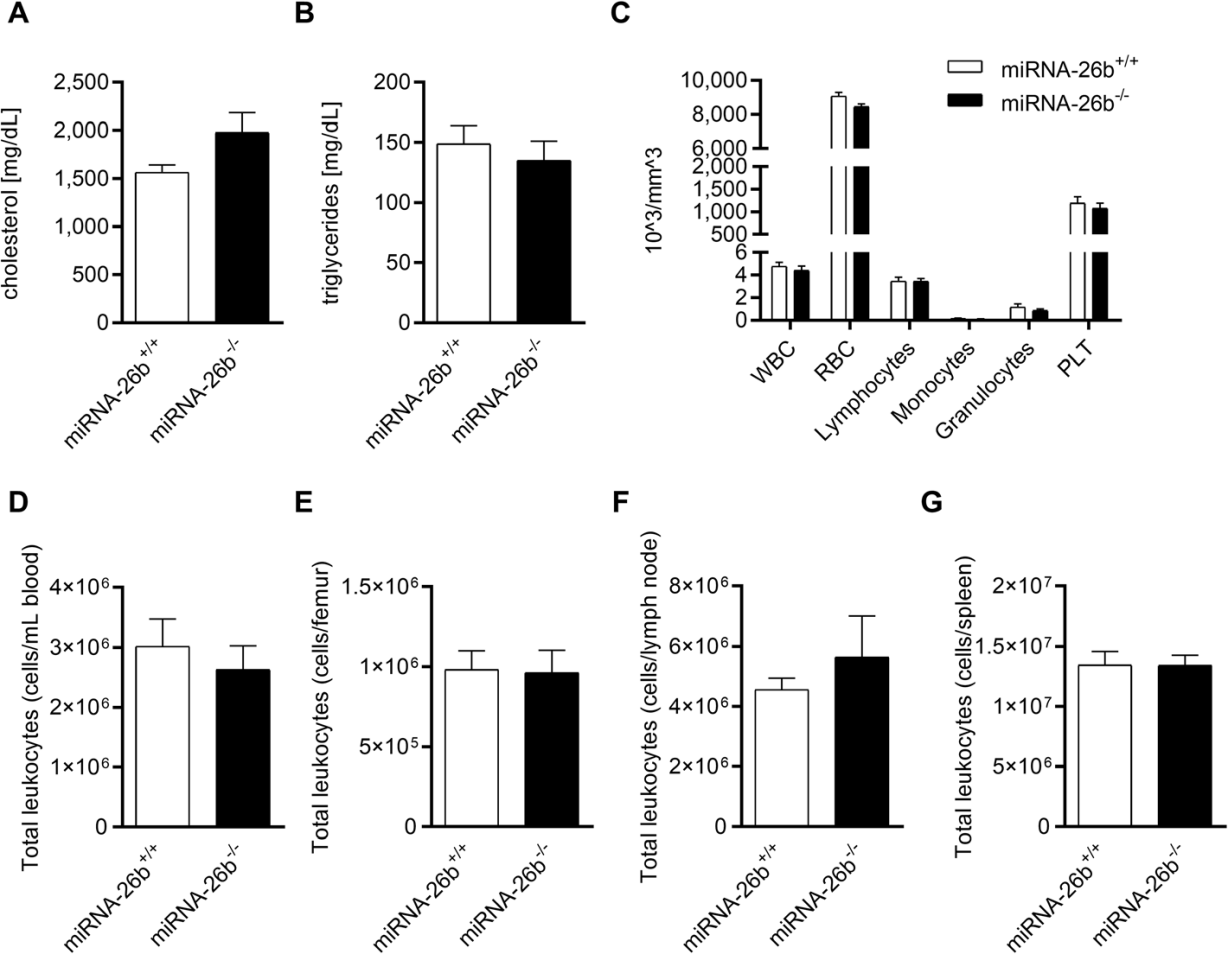
miRNA-26b^−/−^ does not have any effects on physiological parameters. Plasma cholesterol (**a**) and plasma triglycerides (**b**) analysis in the plasma of miRNA-26b^+/+^ and miRNA-26b^−/−^ mice at 8 weeks of age after chow diet (*n* = 3–6). **c** Differential blood count analysis of miRNA-26b^+/+^ and miRNA-26b^−/−^ mice at 8 weeks of age after chow diet (*n* = 6). **d-g** Flow cytometry analysis of CD45^+^ total leukocyte subsets in blood (**d**), bone marrow (**e**), lymph node (**f**) or spleen (**g**) of miRNA-26b^+/+^ and miRNA-26b^−/−^ mice at 8 weeks of age after chow diet (*n* = 9–11). Data represent mean ± SEM, as analyzed by Student t-test/Welch correction or Mann-Whitney test, depending on D’Agostino-Pearson normality testing

Several physiological parameters were analyzed to observe whether the miRNA-26b deficiency has any baseline effects. MiRNA26b^−/−^ mice did not show any obvious morphological or behavioral differences compared to miRNA-26b^+/+^ control mice. Systemic lipid analysis of cholesterol and triglycerides did not show any significant changes upon miRNA-26b deficiency (Fig. 2a-b). Furthermore, general analysis of blood components did not reveal any changes between miRNA26b^−/−^ and miRNA-26b^+/+^ mice (Fig. 2c). Finally, using flow-cytometry analysis it could be demonstrated that miRNA-26b deficiency does not influence total leukocyte counts in important immunological organs, namely blood, bone marrow, lymph nodes and spleen (Fig. 2d-g). More detailed analysis of leukocyte subsets only revealed small changes in neutrophil counts in the blood and spleen and non-classical monocyte counts in the spleen (Tables 1, 2, 3 and 4). Overall, the deficiency of miRNA-26b in mice did, by the currently used methods, not result in significant biological and physiological changes.

**Table 1:** Overview of leukocyte subsets in blood

Blood	***MiRNA-26b***^***+/+***^	***MiRNA-26b***^***−/−***^	***P-value***
Neutrophils [× 10^5^/ml]	9.2 ± 1.7	4.9 ± 0.8	**0.041**
Classical Monocytes [× 10^5^/ml]	1.8 ± 0.3	1.3 ± 0.3	0.205
Non-classical Monocytes [× 10^4^/ml]	8.0 ± 2.5	8.8 ± 1.5	0.794
B cells [× 10^6^/ml]	1.2 ± 0.3	1.2 ± 0.2	0.969
CD3^+^ T cells [× 10^5^/ml]	4.2 ± 0.8	5.1 ± 0.7	0.403
CD4^+^ T cells [× 10^5^/ml]	1.2 ± 0.2	1.4 ± 0.2	0.584
CD8^+^ T cells [× 10^5^/ml]	2.8 ± 0.5	3.6 ± 0.5	0.247

Values (*n* = 10–11) indicate total cell counts per ml of blood, denoted as mean ± SEM, as analyzed by Student t-test/Welch correction or Mann-Whitney test, depending on D’Agostino-Pearson normality testing

**Table 2:** Overview of leukocyte subsets in bone marrow

Bone Marrow	***MiRNA-26b***^***+/+***^	***MiRNA-26b***^***−/−***^	***P-value***
Neutrophils [× 10^5^/femur]	5.7 ± 0.7	5.2 ± 0.8	0.630
Classical Monocytes [× 10^4^/femur]	6.4 ± 1.6	7.1 ± 1.9	0.763
Non-classical Monocytes [× 10^4^/femur]	1.2 ± 0.2	1.8 ± 0.4	0.174
B cells [× 10^5^/femur]	2.2 ± 0.4	2.3 ± 0.3	0.808
CD3^+^ T cells [× 10^4^/femur]	2.5 ± 0.5	2.8 ± 0.8	0.773
CD4^+^ T cells [× 10^3^/femur]	3.8 ± 0.6	3.0 ± 0.5	0.365
CD8^+^ T cells [× 10^3^/femur]	5.6 ± 0.9	6.0 ± 0.8	0.949

Values (*n* = 10–11) indicate total cell counts per femur, denoted as mean ± SEM, as analyzed by Student t-test/Welch correction or Mann-Whitney test, depending on D’Agostino-Pearson normality testing

**Table 3:** Overview of leukocyte subsets in lymph nodes

Lymph Node	***MiRNA-26b***^***+/+***^	***MiRNA-26b***^***−/−***^	***P-value***
Neutrophils [× 10^5^/Lymph node]	4.4 ± 0.2	3.8 ± 0.8	0.080
Classical Monocytes [× 10^4^/Lymph node]	1.6 ± 0.5	1.4 ± 0.4	0.5116
Non-classical Monocytes [× 10^4^/Lymph node]	1.3 ± 0.4	2.2 ± 0.6	0.766
B cells [× 10^6^/Lymph node]	1.5 ± 0.1	1.9 ± 0.4	0.469
CD3^+^ T cells [× 10^6^/Lymph node]	1.6 ± 0.3	2.5 ± 0.6	0.234
CD4^+^ T cells [× 10^5^/Lymph node]	5.1 ± 0.9	7.7 ± 2.0	0.251
CD8^+^ T cells [× 10^6^/Lymph node]	1.0 ± 0.2	1.6 ± 0.4	0.214

Values (*n* = 10–11) indicate total cell counts per lymph node, denoted as mean ± SEM, as analyzed by Student t-test/Welch correction or Mann-Whitney test, depending on D’Agostino-Pearson normality testing

**Table 4:** Overview of leukocyte subsets in the spleen

Spleen	***MiRNA-26b***^***+/+***^	***MiRNA-26b***^***−/−***^	***P-value***
Neutrophils [× 10^5^/Spleen]	11.9 ± 1.0	9.0 ± 0.7	**0.028**
Classical Monocytes [× 10^5^/Spleen]	1.1 ± 0.2	1.0 ± 0.2	0.757
Non-classical Monocytes [× 10^4^/Spleen]	6.5 ± 0.5	18.2 ± 2.6	**< 0.0001**
B cells [× 10^6^/Spleen]	6.2 ± 0.5	6.6 ± 0.5	0.531
CD3^+^ T cells [× 10^6^/Spleen]	2.7 ± 0.2	3.1 ± 0.2	0.294
CD4^+^ T cells [× 10^5^/Spleen]	5.6 ± 0.3	6.6 ± 0.7	0.239
CD8^+^ T cells [× 10^6^/Spleen]	1.7 ± 0.1	2.1 ± 0.2	0.084

Values (*n* = 10–11) indicate total cell counts per spleen, denoted as mean ± SEM, as analyzed by Student t-test/Welch correction or Mann-Whitney test, depending on D’Agostino-Pearson normality testing

### Deficiency of miRNA-26b influences the mouse transcriptome profile

**Fig. 3:**
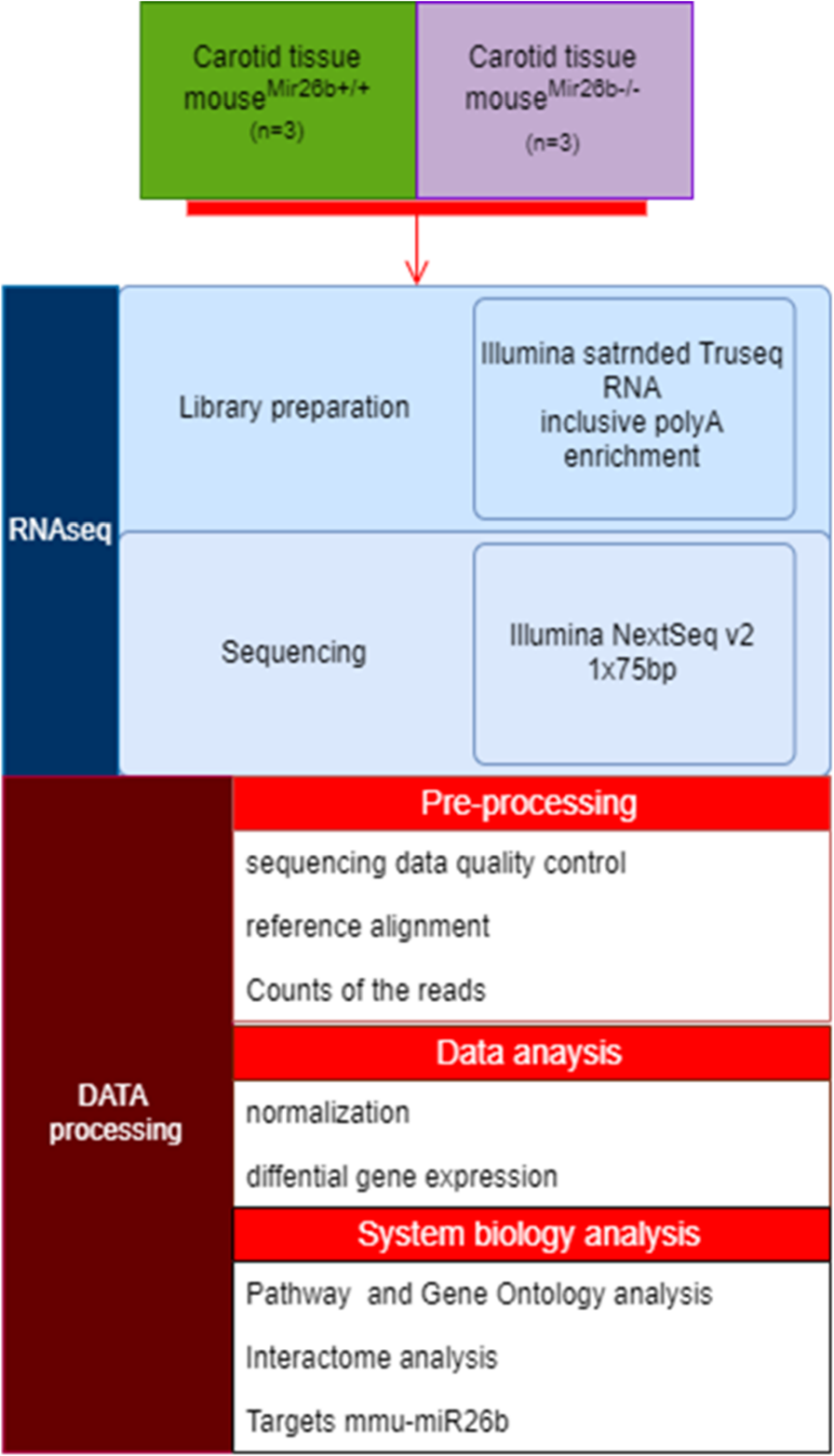
Flowchart NGS analysis. Isolated aortic vessels miRNA-26b^+/+^ and miRNA-26b^−/−^ mice at 8 weeks of age after chow diet (*n* = 3) were used for RNA sequencing (detailed steps are visualized in blue), followed by data processing and analysis (detailed steps are visualized in red)

**Fig. 4:**
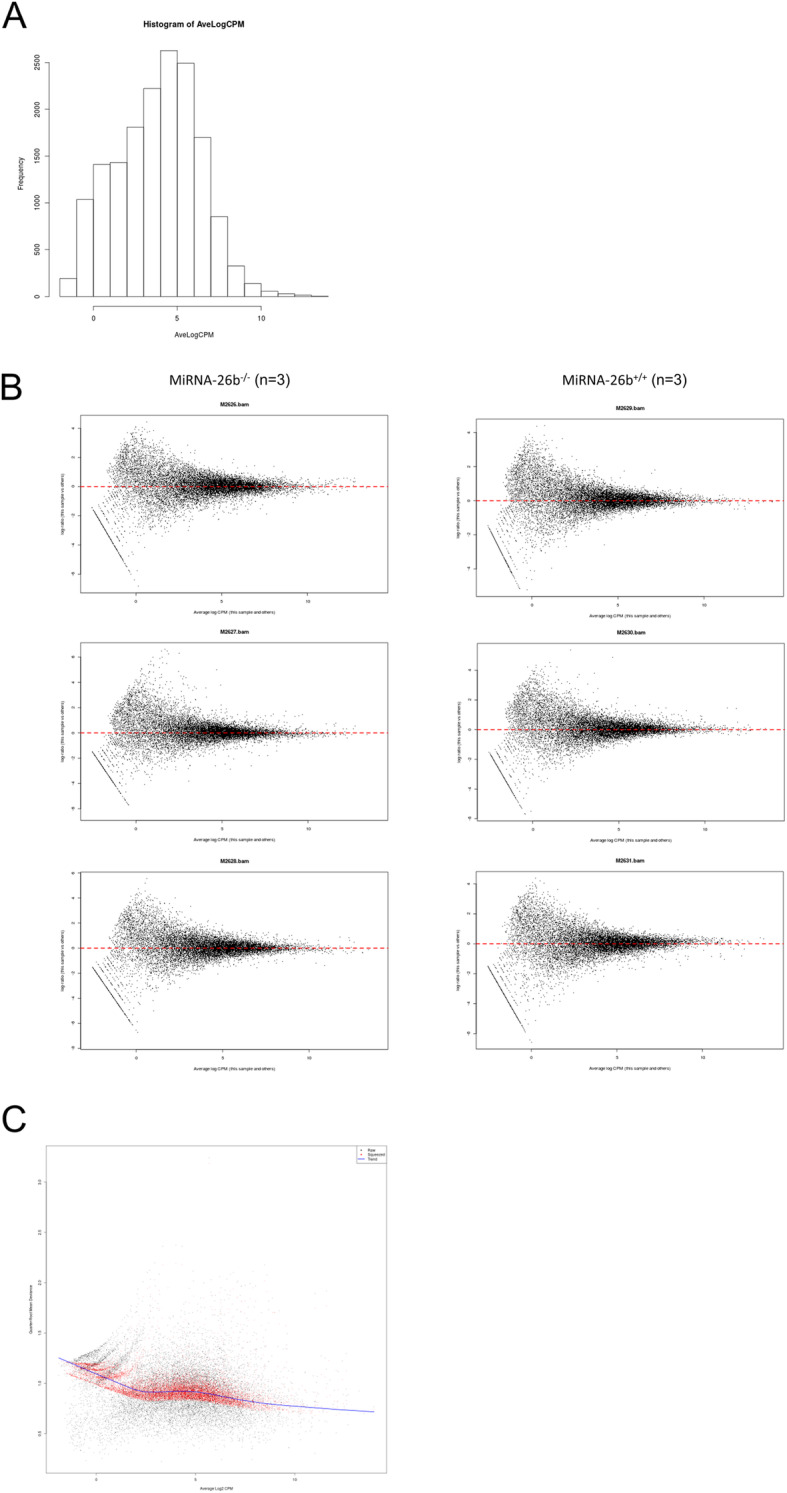
Normalization of RNAseq data. **a** Histogram of average reads count per mega-base (CPM): the data are given as frequencies of Average log read counts per mega-base (AveLogCPM). **b** Single Normalization Summary plots (log-ratios versus log-CPM in all samples) are shown for every sample. **c** AverageLog2 (x-axis) and deviances (y-axis) of each gene are plotted to show that after normalization the trend is maintained; every dot is a gene, in black the genes before normalization, in red the genes after normalization, the blue line represent the trend model

**Fig. 5:**
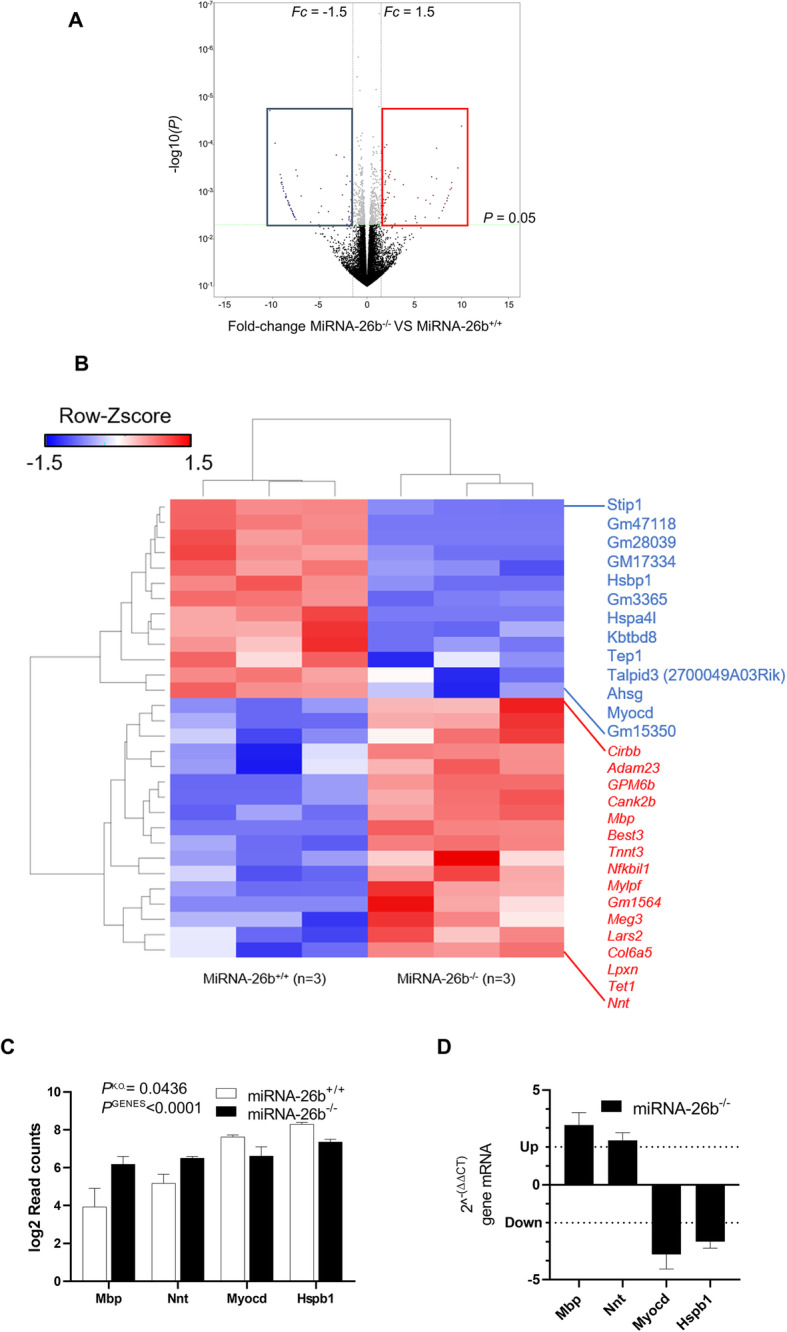
Transcriptome profiling demonstrates significant changes upon miRNA-26b deficiency. **a** ‘Volcano plot’ of statistical significance (P) against fold change (Fc) miRNA-26b^+/+^ and miRNA-26b^−/−^ mice, demonstrating the most significantly differentially expressed genes (P cutoff 0.05, Fc cutoff ≦ − 1.5 or ≧1.5). Every gene is represented by an individual dot. Non-significant genes are visualized in black, significant but not differentially expressed genes in gray, significantly down-regulated genes in blue and significantly up-regulated genes in red. **b** Heatmap showing the top 30 differentially expressed genes, in which each row represents a single gene and each column an individual sample. Down-regulated genes are visualized in blue and up-regulated genes in red. **c** Bar chart showing the top 4 differentially expressed genes for miRNA-26b^−/−^ mice inferred from transcriptomes as log2ReadCounts compared to control mice; *Mbp P* = 0.021128, *Nnt P* = 0.009119, *Myocd P* = 0.024823 and Hspb *P* = 0.000801 by multiple t-test; *P* < 0.0001 for the genes (PGENES), *P* = 0.0436 for miRNA-26b knock down (PKO), by two-way ANOVA with Bonferroni post-test. **d** Bar chart showing the top 4 differentially expressed genes for miRNA-26b^−/−^ mice inferred from 2^-ΔΔ(CT)^ compared to control mice. Data are given as mean ± SEM

In order to validate the mouse model and to further investigate the biological effects of miRNA-26b, aortic vessels from miRNA26b^−/−^ and miRNA-26b^+/+^ mice were subjected to next generation sequencing (Fig. 3) (Gene Expression Omnibus (GEO) DataSets accession ID GSE147519). The data were normalized per library sizes, and genes with low reads count were excluded (sum of counts less than 2) and filtered per count per mega-base (CPM) (Fig. 4). The transcriptome profiling of miRNA-26b^−/−^ mice in comparison to control miRNA-26b^+/+^ mice reveals that over 700 genes are significantly altered (Fig. 5a). Of these, 76 genes were more than 1.5 fold differentially altered and therefore clearly distinguish the miRNA-26b^−/−^ mice from wild type controls. (Fig. 5a). The top 30 differently expressed genes are visualized in a heatmap, in which hierarchical clustering could again clearly demonstrate 2 molecularly distinct clusters confirming the dichotomy between miRNA-26b^−/−^ and miRNA-26b^+/+^ mice (Fig. 5b). To further validate our data-set, we had a closer look at 2 most relevant down-regulated and 2 relevant up-regulated genes (Fig. 5c) and validated these differences independently using PCR analysis (Fig. 5d).

**Fig. 6:**
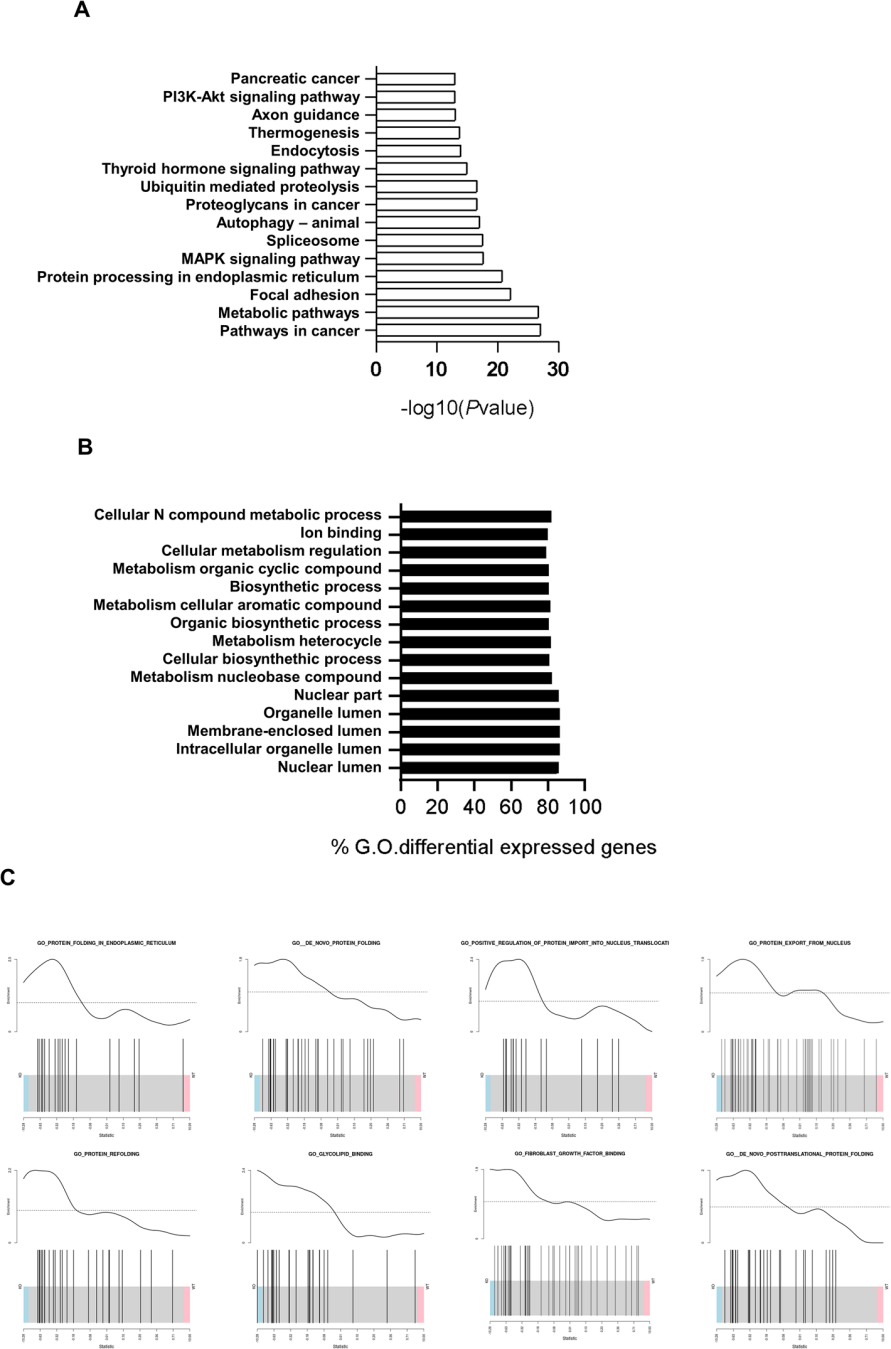
miRNA-26b^−/−^ influences the mouse transcriptome. **a** Bar chart showing the top 15 enriched pathways; data are given as -log10 of statistical significance (−log10*P*). **b** Barchart Top 15 enriched Gene Ontology (G.O) terms; data are given as percentage of genes differentially expressed in the G.O. terms. **c** Gene set Enrichment analysis: for each gene set, the enrichment curve is shown in the top part, whereas the barcode plot is visualized in the bottom part. The barcode plot shows miRNA-26b^−/−^ mice on the left (blue) and miRNA-26b^+/+^ mice on the right (red). Enrichment cutoff was set at 1.3, where 30% of the gene set is enriched

The signature of 766 significantly altered genes was used to examine possible impacts on both pathways and biological processes. This enrichment analysis showed that the overall impact of the miRNA-26b deficiency tends to affect the gene transcription of important homeostatic pathways involved in transcriptional and translational processes linked to several pathologies like cancer, and neurological disorders (Fig. 6a), and Gene ontologies (G.O) in biological processes related to protein biosynthesis and metabolism (Fig. 6b-c).

### MiRNA-26b^−/−^ deficiency differentially influences predicted miRNA-26b target genes

**Fig. 7:**
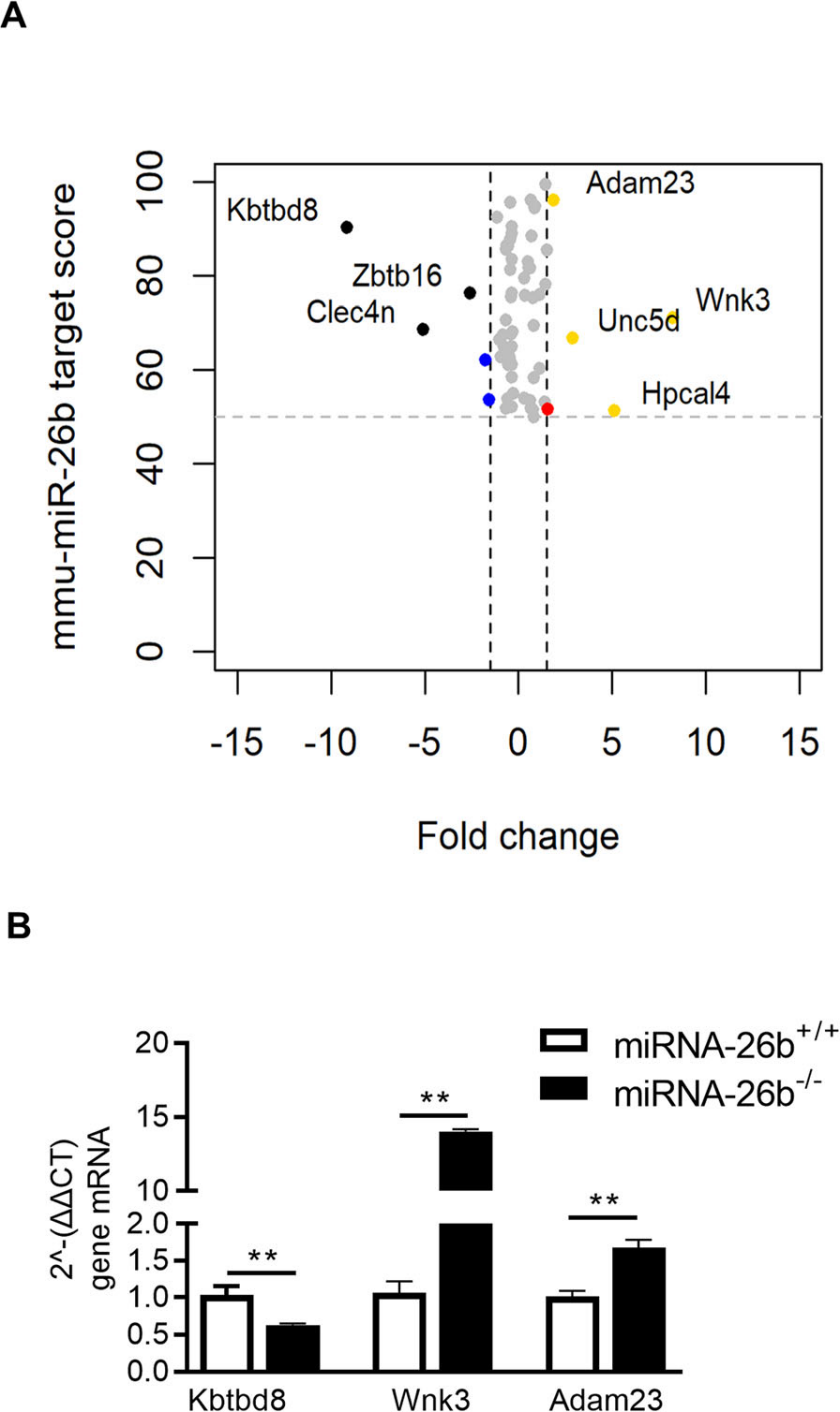
miRNA-26b^−/−^ differentially influences predicted miRNA-26b target genes. **a** ‘Volcano plot’ of miRNA-26b target score (mmu-miRNA-26b target score) against fold change (Fc) between miRNA-26b^−/−^ and miRNA-26b^+/+^ mice, demonstrating the most significantly influenced target genes from miRNA-26b^−/−^. Every target gene is represented by a single dot. Significant but not differentially expressed genes are visualized in gray, significantly down-regulated genes in blue, significantly upregulated gene in red, significantly downregulated genes with high target score in black and significantly upregulated genes with high target score in gold. Mmu-miRNA-26b target score cut-off was set at 50 and Fc at ≦ − 1.5 or ≧1.5. **b** Relative mRNA expression measured by real time PCR. Data represent mean ± SEM, as analyzed by Mann-Whitney test

Finally, we focused our attention on predicted miRNA-26b targets, and evaluated how the miRNA-26b deficiency in our mouse model would affect their level of expression. We retrieved miRNA-26b targets and the relative target prediction scores from miRDB, an online database for microRNA targets and target predictions scores [16, 17]. We focused our analysis only on predicted targets that have a prediction score greater than 50. Of these, 10 transcripts were significantly altered in our data-set. Among these transcripts, 5 were highly up-regulated due to the miRNA-26b deficiency, whereas interestingly 3 of them were highly down-regulated in the sequencing results (Fig. 7a). Real time PCR analysis was performed for 3 of the identified differentially expressed genes, either being the one with the strongest down- or upregulation (*Kbtbd8* and *Wnk3*, respectively) or the highest target score (*Adam23*), clearly validating the observed differences (Fig. 7b).

## Discussion

Over the last years it became clear that miRNA-26b most likely plays an important role in a wide variety of pathologies, like cancer, Alzheimer’s disease, hepatitis and CVDs [6–15]. Although the observed associations were very strong, though often very ambiguous, clear causal relationships have not yet been established. This highlights the need for a proper mouse model enabling the study of the role of miRNA-26b in these different pathologies.

In this manuscript, we have described the generation of miRNA-26b knockout mice and analyzed the physiological phenotype and vascular transcriptome of these mice. It is important to note that our mouse model has a deficiency of both the miRNA-26b-3p as well as the miRNA-26b-5p product, making it at this moment impossible to dissect the exact role of the individual mature products, due to the specificity in the maturation process of each miRNA. Another limitation of our study is that, although the mRNA levels did not change upon deficiency of miRNA-26b, we were unable to determine potential changes in CTDSP1 protein levels in our mice which could be a confounding factor though this appears to be rather unlikely. The deficiency of miRNA-26b did not have any major physiological effects on systemic lipid levels, blood parameters or tissue leukocytes, suggesting that most of the effects of miRNA-26b are rather pathologically induced. The lack of such physiological effects makes this mouse model ideally suited to investigate various pathologies as the confounding factors at baseline are rather limited.

Based on the identified and highlighted targets of miRNA-26b in our mouse model, again clear pathological links can be observed. For example, *Wnk3*, a kinase that plays an role in the regulation of ion transporters in both the kidney and extrarenal tissues [18, 19], has also been shown to play a role in various pathologies, like cancer and to developmental processes like cardiovascular and neuronal development [18]. For example, in a mouse model of ischemic neuroglial injury it was demonstrated that inhibition of *Wnk3* results in an accelerated neurological recovery [20]. All other upregulated genes have also been demonstrated to play a role in neurological processes. For *Adam23* it has been shown that it regulates neuronal differentiation by influencing neural progenitor cells [21]. Additionally, *Unc5d* plays an important role in neocortex development [22], whereas *Hpcal4* is a neural calcium sensor [23]. Moreover, pathway analysis also revealed clear neurological links, as miRNA-26b-5p deficiency has effects on processes like axon guidance. Combined with the studies demonstrating effects of miRNA-26b-5p on neurological pathologies [11, 12], this might suggest that at least some of these effects could be mediated via these identified genes. However, more elaborate studies with this miRNA-26b-deficient mouse model in neurological disease models are needed to further validate this.

Besides these neurological effects, several of the identified genes also play a role in cancer-related processes. For example, *Wnk3* has also been shown to play a role in cell apoptosis and metastasis [24, 25]. On the other hand, *Adam23* has been demonstrated to suppress cancer cell metastasis through the interaction with integrins [26]. Furthermore, such tumor suppressor role has also been demonstrated for *Unc5d* [27]. Again, these effects were also supported by the pathway analysis linking miRNA-26b for example to pancreatic cancer and proteoglycans and other cancer related pathways. Especially in light of the ambiguous results regarding the role of miRNA-26b-5p in cancer [6–10], which at least partly is attributable to the various cancer types that have been investigated, it is of crucial importance to further study the exact and particularly causal role of miRNA-26b and the identified targets in cancer development.

In addition, the identified targets also highlight the potential role of miRNA-26b in other, yet unrecognized, physiological and pathological processes. For example, *Clec4n* has been shown to play a role in allergic airway reaction [28] and in protection against mycobacterial infections [29]. Additionally, *Zbtb16* has been shown to promote white adipogenesis and induce brown-like adipocyte formation [30]. Furthermore, *zbtb16* mediates substrate utilization in brown adipocytes [31]. Pathway analysis also revealed links to thermogenesis and metabolic pathways, further supporting this potential link of such processes with miRNA-26b.

## Conclusions

It has become clear over the last years that miRNAs play an important role in physiology and especially also in various pathologies. One of the miRNAs that has been associated to several pathologies is miRNA-26b, although these interactions are still rather ambiguous and clear causal relationship still need to be proven. Our novel mouse model of miRNA-26b deficiency is an ideal tool to confirm the exact role of this miRNA in all of these pathologies. Initial analysis of our model already confirms several of the pathological associations, as clear links are observed to neurological and cancer processes and even revealed novel potential pathological interactions. All by all, miRNA26b seems to play an important role in even more pathologies than were initially described. It is important to mention that in this study we focused on gene expression changes using transcriptomic analysis, thus only evaluating a subset of the miRNA-26b targets. As miRNAs also play a key role in inhibiting translation, rather than gene expression destabilization, future proteomic analysis would be highly interesting to identify further effects of miRNA-26b deficiency on its target genes/proteins. Furthermore, we focused in our study only on arteries for the moment, a further analysis of other peripheral tissues could provide additional insight into the role of miRNA-26b. Although our mouse model on baseline only presents a small amount of differentially regulated genes, it would be of great importance to evaluate this mouse model in various pathological conditions to evaluate the effect of miRNA-26b on target genes in diseased conditions. Additionally, further studies using the miRNA-26b deficient mouse model are needed to confirm the causal relationship in all these pathologies.

## Methods

### Conditional inactivation of miR26b in mice

The steps leading to the creation of mice carrying the Cre and homozygous floxed miR26b are illustrated in Fig. 1a. We first designed and cloned the targeting vector followed by the generation of founder mice carrying the floxed miR26b gene. The founder mice were mated with Flp and then with Cre mice to generate the F3 KO offspring.

### Construction of the targeting vector

We induced homologous recombination in embryonic stem (ES) cells using a construct containing 2.0 kb of gDNA, including exon 2,3 and 4, as also part of intron 4–5, a Neo Cassette was inserted in between Frt sites. 160 bp of gDNA, including miR26b was flanked by loxP sites (floxed). The construct was finished by introducing a 5.6 kb fragment of gDNA serving as 3′ recombination arm (Fig. 1a). A correctly targeted ES cell clones were injected into blastocysts to produce a chimeric mice, which transmitted the modified allele through the germ line. A male heterozygous for the targeted allele was bred with a female expressing ubiquitous Flippase (Flp) transgene to ultimately produce animals that had deleted the Neo cassette, preserving the loxP sites flanking exon miR26b.

### ES-cell electroporation

R1/E (129/Sv) embryonic stem cells (ES), were cultured on irradiated mouse embryonic fibroblasts (MEFs) using the following ES-medium Knock-Out-DMEM, 12% fetal calf serum [FCS], 2 mM L-glutamine, 1 mM sodium pyruvate, 1% penicillin/streptomycin, 100 μM nonessential amino acids (all Invitrogen), 100 μM b-mercaptoethanol) containing leukemia inhibitory factor (LIF). ES cells were electroporated using a BIORAD electroporator (250 V, 500 μF) with 25 μg linearized constructs (pKO-miR26b) and selected with 200 μg/ml Geneticine (G418).

### ES-cell injection

miRNA-26b^−/−^ mice were generated by Laser assisted (XY-Clone Hamilton Thorne) injection of R1/E(129/Sv) cells into 8-cell stage C57Bl/6NCrl embryos. All manipulations were done in the Transgenic Core Facility (TCF) of the MPI-CBG, Dresden. Chimaeras were crossed to C57Bl/6NCrl mice and their offspring was screened for germline transmission. Embryo donor and recipient mice: C57BL/6NCrl, Crl:CD1(ICR) mice were purchased from Charles River. All mice were housed in IVC units and maintained on a 12-h dark/12-h light cycle. For the production of eight cell stage embryos, 8-week old females were natural mated. The females were screened for vaginal plugs the next morning (0.5 days post coitum, dpc) and housed until embryos were collected by day 2,5 dpc. Crl:CD1(ICR) females were mated with vasectomized Crl:CD1(ICR) males and used as recipients for injected embryo transfer at 0.5 dpc into the oviduct.

### Southern blot

Southern blot was performed as described in DecaLabel™ Biotin DNA kit (Thermo Fisher K0651) The gDNA was cut by BspHI and probed by 0.7 kb amplified fragment binding upstream of 5′ recombination arm.

### Primer design and genotyping of miR26b KO mice

Genotyping of the animals was performed by conventional PCR. The gDNA was isolated by Trizol (Thermo Fisher CatNr: 15596026) using mouse tail biopsies. We set primer mix 1 and 2 and primer mix 1 and 3. Primer 1- (3′ out of miR 26b) – 5′-CATTCGCTGTAGGAACTCATCTAC-3′; Primer 2- (5′ out of miR 26b)-5′-GAGTGAGAACCCAAGACTACCTG-3′; Primer 3-(in miR26b)-5′-GACCCAGTTCAAGTAATTCAGG-3′. All PCR reactions were amplified with GoTaq (Promega M7808).

### Real-time PCR

For RNA analysis, total RNA was isolated from mouse aortas and reverse-transcribed into cDNA using Mo-MLV RT (Invitrogen). RT-PCR was performed using TaqMan Gene Expression Master Mix and Real time specific primer pairs (Applied Biosystems). All reactions were run on a 7900HT thermocycler (Life Technologies GmbH), essentially as described [32]. The expression levels of the target genes were quantified by the ratio to 18S RNA levels, detailed information is given in the table below. The results are reported as relative gene expression (2^-ΔΔCT^) or undetermined when the amplification is not detected by Relative Quantification Manager software (Life Technologies GmbH).

**Table:** 

Gene	Company	Catalog Number
18S RNA	Thermo Fisher	4352930E
miR26b-3p	Thermo Fisher	002444
miR26b-5p	Thermo Fisher	000407
miR26a-1	Thermo Fisher	002443
miR26a-2	Thermo Fisher	463,227 mat
Myocd	IDT	Mm.PT.58.5756972
Mbp	IDT	Mm.PT.58.13897707
Nnt	IDT	Mm.PT.58.50497613
Hspb1	IDT	Mm.PT.58.41302123
Ctdsp1	IDT	Mm.PT.58.41127385
Adam23	IDT	Mm.PT.58.30118948
Wnk3	IDT	Mm.PT.58.32266018
Kbtbd8	IDT	Mm.PT.58.5701424

### Mice

All mice (in house creation as described above) were on the C57BL/6 background and were kept in a SPF facility with normal light/dark cycles and ad libitum access to food and water. Animals were fed a normal laboratory diet (Sniff V1534–300) and randomly allocated to experimental groups. miRNA-26b^−/−^ mice were derived after intercrossing with CreERT2 mice and deleting the allele by tamoxifen injection. The CreERT2 gene was eliminated by breeding. Animals are euthanized under a deep, non-antagonisable anaesthetic (6–8 mg/kg xylazine and 90–120 mg/kg ketamine) with subsequent blood collection by retroorbital puncture using anticoagulant capillaries.

### Lipid and blood count analysis

Cholesterol and triglyceride levels were quantified in EDTA (ethylenediaminetetraacetic acid)-buffered plasma using an enzymatic assay (Roche) according to the manufacturer’s protocol. Freshly obtained EDTA blood was used to analyze leukocyte counts using an animal blood counter (scil Vet ABC Hematology Analyzer).

### Flow cytometry

Whole blood obtained was collected in EDTA-buffer tubes. Samples were subjected to red-blood-cell lysis for further analysis using flow cytometry. Bone marrow cells were harvested by flushing femurs with Hank’s Medium (Hanks’ Balanced Salt Solution + 0.3 mmol/l EDTA + 0.1% BSA) (Gibco by life technologies). Spleen and lymph nodes were mechanically crushed and passed through a 30 μm cell strainer (Cell-Trics, Partec) using Hank’s Medium and to obtain single cell suspensions. Single cell suspensions were subsequently stained with different antibody cocktails and analyzed using a FACS Canto II, using the FACSDiva software (BD Biosciences). Cell populations were discriminated by the following antibody cocktail: anti-CD45, anti-CD115, anti-Gr1, anti-CD11b, anti-B220, anti-CD3, anti-CD4, anti-CD8 (All eBioscience). Cell populations and marker expression were gated as depicted below using the FlowJo analysis program (Treestar): neutrophils (CD45^+^CD115^−^Gr1^high^), classical monocytes (CD45^+^CD115^+^Gr1^high^), non-classical monocytes (CD45^+^CD115^+^Gr1^low^), B-cells (CD45^+^CD115^−^Gr1^−^B220^+^), CD3^+^T-cells (CD45^+^CD115^−^Gr1^−^CD3^+^), CD4^+^T-cells (CD45^+^CD115^−^Gr1^−^CD3^+^CD4^+^) and CD8^+^T-cells (CD45^+^CD115^−^Gr1^−^CD3^+^CD8^+^).

### Sample preparation for RNA-sequencing

After sacrifice (see Mice for more details), mice were perfused with phosphate buffered saline and subsequently the aortic arch and thoracic aorta was explanted, collected in RNA-later solution and snap frozen in liquid nitrogen.

Total RNA isolations were done at Microsynth, Switzerland. In brief, total RNA was isolated using the RNeasy Mini Kit (Qiagen, Hombrechtikon, Switzerland) following the supplier instructions. The total RNA was quantified using RiboGreen® and the RNA quality was check using the Bioanalyzer® platform (RIN value) before library preparation. The libraries were prepared according to TruSeq RNA Library Prep Kit v2 (Illumina, San Diego, California, U.S) specifications, and then sequenced by Illumina HiSeq 2000 with a 30 million reads depth.

### Sequence alignment, differential expression and enrichment analysis

RNAseq sequencing files were aligned with GRCm38/mm10 version using STAR. The transcripts abundances were estimated with the package Subread2 using the FeatureCounts function running on Unix environment using as gene-annotation database the gene-code Release M23 (GRCm38.p6). The data were filtered, normalized and the differential gene expression was computed using the packages limma (https://bioconductor.org/packages/release/bioc/html/limma.html) and edger (https://bioconductor.org/packages/release/bioc/html/edgeR.html) on R. The pathways enrichment and the gene sets enrichment were assessed with Camera package on R3.

### Generation of plots

All heatmaps were created using the statistical computing language R (http://www.R-project.org) using the heatmap.2 function in the gplots package (http://cran.r-project.org/web/packages/gplots/index.html); volcano plots were generated with the package EnhancedVolcano (https://github.com/kevinblighe) Barcharts were created using Graph Prism v8.0 (GraphPad, San Diego, California).

### Data deposition

Raw .bam files, and processed data are deposited on Gene expression omnibus (https://www.ncbi.nlm.nih.gov/geo) with the series number GSE147519.

### Statistical analyses

For mouse phenotyping, statistical calculations were performed using GraphPadPrism (GraphPad Software Inc.). Normality was tested using the D’Agostino and Pearson omnibus K2 normality test. Data that did not pass the normality test were further analyzed using the Mann-Whitney test. Normally distributed data are analyzed using the unpaired t-test with Welch correction. All data are presented as mean ± SEM.

## Supplementary Information

**Additional file 1.**

## Data Availability

All datasets used and/or analysed during the current study are available from the corresponding authors on reasonable request. The raw data from the next-genome sequencing has been deposited at Gene expression omnibus (https://www.ncbi.nlm.nih.gov/geo/query/acc.cgi?acc=GSE147519).

## Abbreviations

AveLogCPM: Average log read counts per mega-base
CPM: Count per mega-base
CT: Cycle threshold
CVD: Cardiovascular disease
EDTA: Ethylenediaminetetraacetic acid
ES: Embryonic stem
FC: Fold change
FCS: Fetal calf serum
Floxed: Flanked by loxP sites
Flp: Flippase
GEO: Gene expression omnibus
G.O.: Gene ontologies
LIF: Leukemia inhibitory factor
MEF: Mouse embryonic fibroblast
miRNA: MicroRNA
UTR: Untranslated region
