# Correction to: Transcriptome signature of miRNA-26b KO mouse model suggests novel targets

**DOI:** 10.1186/s12863-021-00990-3

**Published:** 2021-09-20

**Authors:** Emiel P. C. van der Vorst, Mario A. A. Pepe, Linsey J. F. Peters, Markus Haberbosch, Yvonne Jansen, Ronald Naumann, Georgios T. Stathopoulos, Christian Weber, Kiril Bidzhekov

**Affiliations:** 1 Institute for Cardiovascular Prevention (IPEK), Ludwig-Maximilians-Universität (LMU) München, Munich, Germany; 2 DZHK (German Centre for Cardiovascular Research), Partner site Munich Heart Alliance, Munich, Germany; 3 Interdisciplinary Center for Clinical Research (IZKF), Institute for Molecular Cardiovascular Research (IMCAR), RWTH Aachen University, Aachen, Germany; 4 Department of Pathology, Cardiovascular Research Institute Maastricht (CARIM), Maastricht University, Maastricht, the Netherlands; 5 Helmholtz Center Munich, Munich, Germany; 6 MPI of Molecular Cell Biology and Genetics, Dresden, Germany; 7 Department of Biochemistry, Cardiovascular Research Institute Maastricht (CARIM), Maastricht University, Maastricht, The Netherlands; 8 Munich Cluster for Systems Neurology (SyNergy), Munich, Germany

**Correction to: BMC Genom Data 22, 23 (2021)**

**https://doi.org/10.1186/s12863-021-00976-1**

Following publication of the original article [1], the authors identified an error in the author name of Ronald Naumann.

The incorrect author name is: Ronald Nauman

The correct author name is: Ronald Naumann

The author group has been updated above and the original article [1] has been corrected.
