# Supplementary figures and images for: Transcriptome signature of miRNA-26b KO mouse model suggests novel targets
Source: BMC Genom Data. 2021 Jun 30;22:23. doi: 10.1186/s12863-021-00976-1 (PMC8243710; doi:10.1186/s12863-021-00976-1)

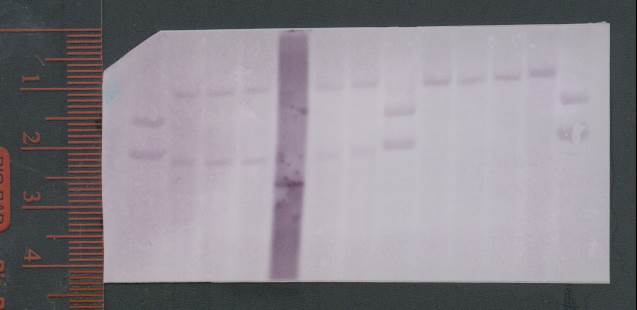

Supplement: Supplementary file 1 — Additional file 1. [file 12863_2021_976_MOESM1_ESM.zip › Van der Vorst et al._Additional file 1_Raw image 1B.jpg]

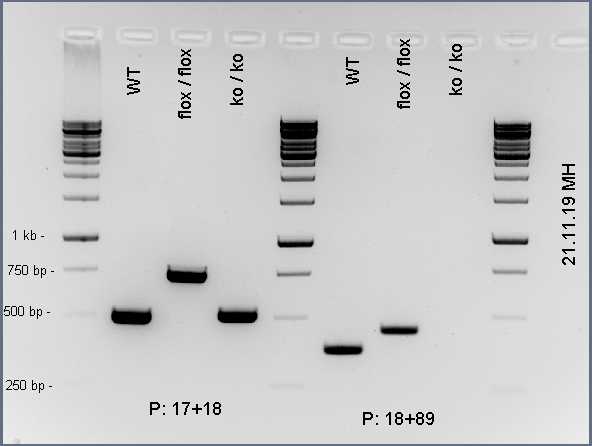

Supplement: Supplementary file 1 — Additional file 1. [file 12863_2021_976_MOESM1_ESM.zip › Van der Vorst et al._Additional file 1_Raw image 1C-D.jpg]
